# Transverse mode coupling in monolithic few-mode fiber laser oscillators

**DOI:** 10.1038/s41377-025-01862-6

**Published:** 2025-05-12

**Authors:** Binyu Rao, Jinbao Chen, Zefeng Wang, Hao Li, Baolai Yang, Rong Zhao, Xinyu Ye, Hengyu Tang, Meng Wang, Zhixian Li, Zilun Chen, Jianqiu Cao, Hu Xiao, Wei Liu, Pengfei Ma, Tianfu Yao

**Affiliations:** 1 College of Advanced Interdisciplinary Studies, National University of Defense Technology, Changsha, China; 2 Nanhu Laser Laboratory, National University of Defense Technology, Changsha, China

**Keywords:** Fibre lasers, Nonlinear optics

## Abstract

Transverse mode instability (TMI), induced by nonlinear thermal-optical coupling, poses a primary challenge for the power scaling of fiber lasers. In the fiber oscillator, a sealed resonant cavity, TMI could become particularly complex due to the mode competition during the laser oscillation. While traditional theories of TMI predominantly address two-mode coupling, this paper explores the TMI phenomena in few-mode fiber oscillators utilizing a holistic approach that includes solving steady-state thermal-optic coupling equations. The simulation shows that there is a non-monotonic correlation between bending loss and the TMI threshold, which is contrary to the monotonic associations suggested by two-mode interaction theory. When one high-order mode experiences net gain, fluctuations of the TMI threshold would occur, leading to the amplification of a new mode within the uncoupled frequency region, thus affecting the gain saturation. By designing the linewidth of a low-reflection grating (LR), the modal power management in the uncoupled frequency domain can be achieved. An excessively broad LR linewidth exacerbates mode coupling within the shared frequency region, thus exacerbating TMI. To validate the theoretical simulation, we carefully fabricated LRs and optimized the fiber coiling to elevate the TMI threshold. Through careful optimization of LR linewidth and bending radii, we achieved a record-breaking laser output of 10.07 kW using a monolithic fiber oscillator, with no observable evidence of TMI. Our work demonstrates that modal power redistribution in independent frequency domains offers a novel approach to mitigating TMI in high-power fiber lasers. Additionally, it provides new insights into mode decoupling strategies pertinent to fiber communications.

## Introduction

High-power fiber lasers are widely employed as laser sources in industrial processing and scientific research, due to the advantages of high beam quality, high efficiency, simple thermal management, and transmission flexibility. The fiber laser oscillator with its monolithic resonant cavity offers resistance to environmental interference, superior robustness, and simple control logic. Over the past decade, fiber lasers have made remarkable progress in power scaling. There have been several reports of 5–8 kW level single-stage monolithic fiber laser oscillators^1–5^, which employed various resonant cavity designs to mitigate the power scaling limitations. Despite these achievements, critical challenges persist in the fiber laser development, including nonlinear effects represented by stimulated Raman scattering (SRS) and transverse mode instability (TMI) induced by thermal-optical effects. Many feasible schemes^6–9^ could be used to suppress nonlinear Raman components. While multiple strategies have been proposed to suppress nonlinear Raman components, TMI remains a more formidable obstacle, due to a sudden onset of beam break-up and the rapid degeneration of beam quality^10^. The TMI-induced transferring of mode energy in fiber laser generally originates from thermal effect^11–15^, photodarkening^16,17^, and nonlinear effect-induced mode degeneration^18–22^. The TMI dynamics are also influenced by the factors such as driver noise^23^, polarization state^24^, pump configuration^25–27^, fiber design^28–31^ and fiber coiling^32,33^.

There have been studies on TMI in the fiber laser oscillator in the last years^34–36^, which mainly focused on nearly single mode operations. However, in the large-mode-area ytterbium-doped fibers, few-mode operation is common in the absence of effective mode selection techniques. Consequently, the gain competition between modes also significantly impacts TMI in the few-mode fiber oscillator. Moreover, in the few-mode operation, the mode competition becomes more pronounced in the fiber oscillator, leading to a significant influence on the transfer of mode energy. In fact, reports of theoretical and experimental studies on TMI in the few-mode fiber oscillator are rare. In 2020, Yun Ye investigated the TMI issue in a bidirectional-pumped few-mode all-fiber oscillator. The TMI threshold was enhanced to ~3.6 kW by decreasing the core-to-cladding ratio^37^ of the gain fiber. In 2022, our group mitigated TMI by optimizing the pump wavelength and achieved an 8-kW-level fiber oscillator employing femtosecond-laser written fiber Bragg gratings (FBGs)^38^. Published theoretical researches mainly focus on the TMI in the two-mode fiber laser oscillator^34,36^, neglecting other higher-order modes. The limitation restricts the ability to accurately analyze TMI threshold evolution in few-mode fiber laser oscillators.

In this manuscript, we presented a theoretical study of TMI in the few-mode all-fiber oscillator. By using steady-state thermal-optical coupling equations, combined with multimode steady-state rate equations, the mode competition and energy transfer could be clearly described by the calculated evolution of different mode components. When a high-order mode has large-mode loss, it struggles to sustain itself in the fiber oscillator. Under such conditions, the gain of the high-order mode mainly comes from the TMI-induced energy transfer. Consequently, the TMI threshold variation of the few-mode oscillator shows a similar drop-off to that of the near-single-mode fiber laser as the bend radius increases. When one high-order mode has a net gain in the cavity, it will be amplified at one frequency region, uncoupled from other modes, thanks to the modal frequency selection characteristics of FBGs. As each mode transitions from negative gain to net gain, the TMI threshold is expected to fluctuate between regions of decreasing and increasing mode loss. Therefore, to achieve higher total power, it is essential to select an appropriate bend loss, which ensures that modal energy is orderly distributed into uncoupled frequency components. However, if a low-reflection grating (LR) with a larger linewidth is employed, the frequency regions of different modes could overlap, leading to possible coupling in the overlapped frequency regions and a consequent reduction in the TMI threshold.

Based on the theoretical analysis, experimental researches are conducted to evaluate the effects of the linewidth of LR and bending radius on TMI in a few-mode fiber oscillator. The experimental results demonstrate that both LR linewidth and bending radius must be carefully optimized to achieve an acceptable TMI threshold. By optimizing both the LR linewidth and the bending radius, we successfully achieved a stable laser output of 10.07 kW with no evidence of TMI in an all-fiber oscillator. To the best of our knowledge, it represents the highest power to date achieved by using a single-stage monolithic fiber laser oscillator setup. We demonstrate that TMI mitigation through the modal power redistribution in the uncoupled frequency domain, both theoretically and experimentally examined in this study. This approach reveals an unsettled variation in TMI threshold, which could offer fresh insights and strategies for mitigating TMI in high-power fiber lasers and contributing to a deeper understanding of the TMI theory under the condition of multimode excitation.

## Results

### Theoretical analysis

The numerical simulations were performed to investigate the thermally-induced mode coupling in the few-mode fiber oscillator. The thermally-induced mode coupling was characterized by the photothermal coupling equation^12,13,15^, and details of the theoretical model are outlined in “*Materials and methods*”.

**Fig. 1:**
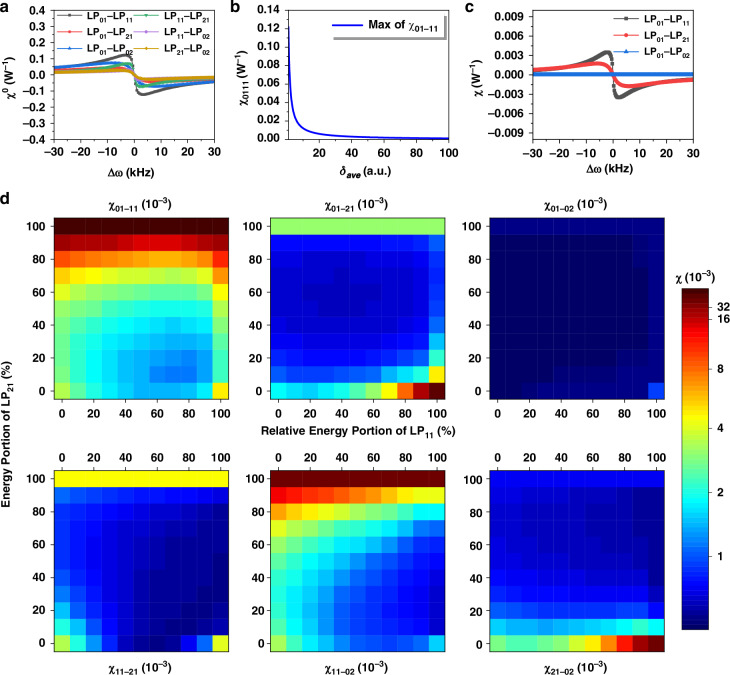
**Thermally-induced coupling coefficients between different modes.** **a** Coupling coefficients under uniform-gain condition vary with coupling frequency: ${{\rm{\chi }}}_{01-11}^{0}$ (black line), ${{\rm{\chi }}}_{01-21}^{0}$ (red line), ${{\rm{\chi }}}_{01-02}^{0}$ (blue line), ${{\rm{\chi }}}_{11-21}^{0}$ (green line), ${{\rm{\chi }}}_{11-02}^{0}$ (purple line), ${{\rm{\chi }}}_{21-02}^{0}$ (brown line). **b** Coupling coefficient vary with coupling frequency considering gain saturation with average power: ${{\rm{\chi }}}_{01-11}$ (black line). **c** Coupling coefficients vary with coupling frequency setting gain saturation as that where LP_01_ occupying the most proportion. **d** Coupling coefficients vary with different modal proportion. The relative energy portion of LP_11_ (*x* axis) represents the portion of LP_11_ in the energy after removing portion of LP_21_ (*y* axis)

Under idealized conditions, assuming a uniform-gain profile and neglecting the gain variation along the *z* axis, the thermally-induced coefficient χ_0_, defined as the overlap integral covering two coupling mode fields and the induced thermal source field, is calculated. The results are shown in Fig. 1a. The coefficient with the highest numerical value is ${{\rm{\chi }}}_{01-11}^{0}$ followed by ${{\rm{\chi }}}_{11-21}^{0}$ and ${{\rm{\chi }}}_{01-02}^{0}$. However, in practical settings, the gain profile can be significantly influenced by the gain saturation effect^39^. Incorporating the gain saturation, the thermally-induced coefficient ${{\rm{\chi }}}_{01-11}$ is calculated, as illustrated in Fig. 1b. Notably, the thermally-induced coefficient drops rapidly with an increase of the gain saturation coefficient^39^ *δ*_*ave*_, which quantifies the contribution of average power to the gain saturation.

Under more realistic conditions, the gain saturation coefficient is considered to vary according to the transverse distribution of laser intensity, which causes the thermally-induced coefficient associated with the different modal components in the laser profile. For example, when LP_01_ mode dominates in the laser intensity *I*_*s*_(**r**), the gain saturation coefficient *δ*_*dis*_(**r**), which is used to descried the degree of the gain saturation varied with the transverse distribution of laser intensity, typically exhibits a large value in the center position of fiber core for the excessive consumption of excited particles and has a small value at the core edge owing to the redundancy of excited particles, suppressing the modal coupling that shares the angular distribution of the LP_01_ mode. Figure 1c illustrates the calculated thermally-induced coefficients under pure LP_01_ excitation, revealing that the numerical magnitude between ${{\chi }}_{01-21}$ (red line) and ${{\chi }}_{01-02}$ (blue line) differs from the uniform-gain case depicted in Fig. 1a. Indeed, the dominance of LP_01_ component in the gain saturation suppresses coupling between LP_01_ and LP_02_ as ${{\chi }}_{01-02}$ is much smaller in this case.

As displayed in Fig. 1d, the coupling coefficients under various mode proportions are shown, considering three modes: LP_01_, LP_11_, and LP_21_. Comparing heat maps reveals that, in most cases, ${{\chi }}_{01-11}$ is typically the maximal coefficient indicating the strongest coupling between LP_01_ and LP_11_. However, an increase in the proportion of LP_11_ will enhance the coupling coefficient between other modes, suggesting that the relatively strong coupling can also occur between LP_01_ and LP_21_, as well as between LP_11_ and LP_21_. During coupling, the gain saturation acts as a mediator, when a particular fiber mode commands a relatively high proportion of laser power, its associated coupling will diminish, whereas coupling among other modes would intensify. This phenomenon also exists in the gain competition among various fiber modes. Consequently, the variations in coupling coefficients due to gain saturation would be manifested as the fluctuation of TMI threshold of the fiber laser.

**Fig. 2:**
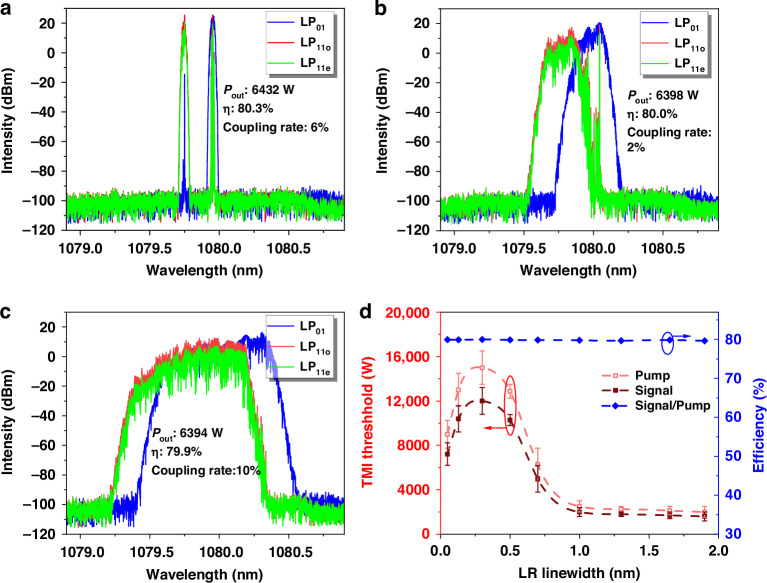
**The simulated results of spectra and the variation in TMI threshold corresponding to different LR linewidths.** **a** Spectra of oscillator with LR linewidth of 0.05 nm. **b** 0.3 nm. **c** 1.0 nm, and **d** variation of TMI threshold with the switching of LRs with different linewidth. Pump power at TMI threshold (right *y* axis) is denoted by pink dots and is represented by a pink dashed line and signal power is denoted by dark red dots and is represented by dark red dashed line both including their respective error bars. Efficiency is plotted as blue dots and fitted as blue dashed line (left *y* axis)

Then, incorporating the calculated thermally-induced coupling coefficients into the thermal coupling term and establishing the modal frequency response to FBGs as the boundary conditions (details are provided in “*Materials and methods*”), enables the acquisition of the simulation results for the properties of the oscillator laser. Considering a specific condition with a 10 cm bending radius, three modes, namely LP_01_ and LP_11e/o_, are predicted to oscillate in the oscillator. Figure 2 presents the corresponding results under various linewidth settings of the LR. The simulated spectra of signal laser with different linewidths at signal power of ~6400 W are depicted in Fig. 2a, c. When LR is narrow, with the 3-dB linewidth of 0.05 nm, nondegenerate modes clearly exhibit individual resonant peaks. Minor spectral components of LP_11e/o_/LP_01_ mode can also be observed at the respective primary resonant peaks of LP_01_/LP_11e/o_, indicating the thermally-induced mode coupling. As LR widens to 0.3 nm, the resonant peaks broaden their linewidths, accompanied by decreases in spectral power density, which would lead to the weakening of the component of thermally-induced mode coupling. With the further widening of the LR to a 1.0 nm linewidth, most of the spectral components of the resonant peaks become overlapped, where the mode coupling would be exacerbated. Here, the TMI threshold is defined as the signal power when coupled mode power exceeds 5% of the total power. By setting different LR linewidths, the variations of TMI threshold are simulated and displayed in Fig. 2d. Based on the simulated results, it can be concluded that LR linewidth should be optimized to achieve a relatively high TMI threshold.

Having explored the effect of linewidths of modal spectral responses on TMI in the few-mode fiber oscillator, we investigate the influence of modal loss on thermally-induced coupling. By examining the role of bending loss, associated with different core modes in shaping the thermal profile of the oscillator, we aim to fully assess how these losses contribute to or mitigate the thermal coupling effect.

**Fig. 3:**
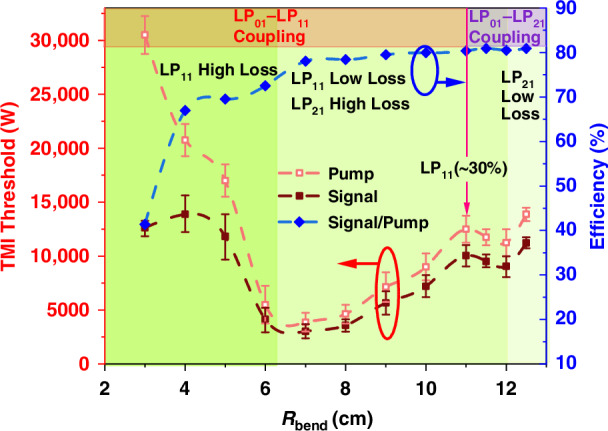
**Variation in the TMI threshold of a few-mode fiber oscillator associated with the change of the bending radius.** Throughout the initial stage and a considerable portion of the second stage, coupling between LP_01_ and LP_11_ is predominant (indicated by the red region). Once the portion of LP_11_ exceeds 30%, the stronger coupling would occur between LP_11_ and LP_21_ (highlighted by the purple region)

Based on the calculated mode bending loss, as detailed in “*Materials and methods*” section, and thermally-induced coefficient, the TMI threshold of a few-mode oscillator with varying bending radii is simulated. As shown in Fig. 3, the threshold of TMI varies with the increasing of the bending radius (R_bend_). From the variation curves, the conclusion could be drawn that the TMI threshold has a nonmonotonic variation as the bending radius increases. As the bending loss decreases, the oscillator goes through three stages.

In the first stage, the bending loss for higher-order modes is significantly greater than that for the fundamental mode, leading to the fundamental mode dominating the laser output power. In the near-single-mode regime, the TMI threshold gradually decreases as the bending loss of LP_11_ mode diminishes, consistent with the conventional theory.

In the second stage, the bending loss of LP_11_ mode decreases to an extent that LP_11_ mode acquires net gain and oscillates in the cavity. As LP_11_ mode gradually occupies a certain share of the total laser power, the ${\rm{\chi }}$_0111_ diminishes due to gain saturation, thereby elevating the TMI threshold.

In the third stage, the LP_11_ mode takes a larger portion of the total power (>30%), and the ${\rm{\chi }}$_0121_ emerges as the maximum value among the coupling coefficient, indicating that the strongest coupling occurs between LP_01_ and LP_21_ mode. Consequently, as the bending loss of LP_21_ mode decreases, the TMI threshold decreases. However, once the bending loss of the LP_21_ mode is sufficiently low to allow it to survive in the cavity and take up a certain portion, the coupling between LP_01_ and LP_21_ mode would be suppressed by gain saturation, and the TMI threshold would again see a growth. This outcome diverges markedly from the predictions of two-mode TMI theory. Collectively, by controlling the mode loss, one can effectively redistribute the power among various core modes into separate, uncoupled frequency components, thus facilitating the suppression of TMI.

### Experimental validation

In the theoretical analysis, the TMI threshold of a few-mode fiber oscillator is estimated by integrating the calculated modal frequency response to FBGs, bending loss, and coupling coefficient into and conducting the thermally light coupling power equations. The results suggest that both the LR linewidth and the bending loss have the optimal values for achieving a laser output with relatively high efficiency and TMI threshold. In this part, the experiments are designed to identify the suitable LR linewidth and bending loss for a practical fiber oscillator.

**Fig. 4:**
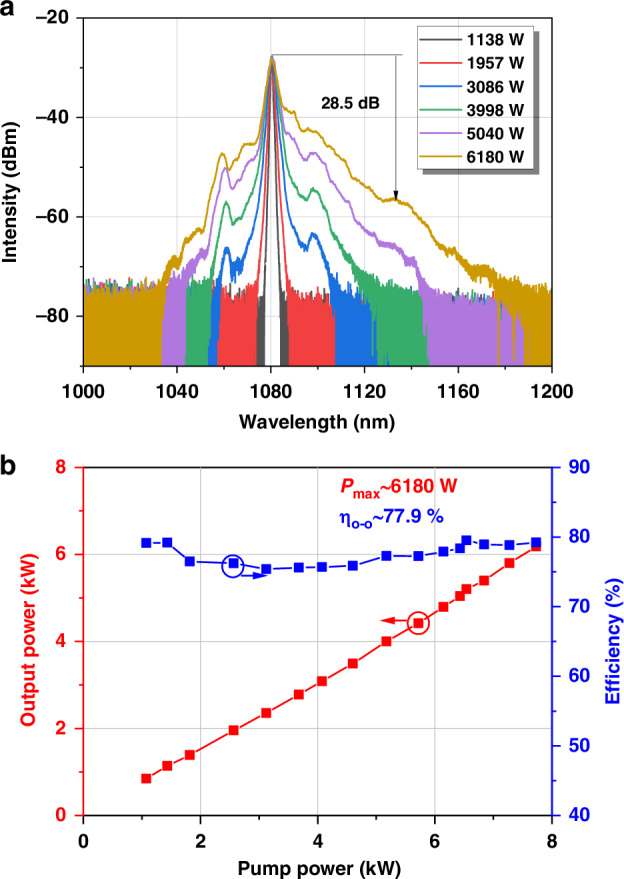
**The performance of the fiber oscillator with 0.2-nm LR.** **a** Spectra across varying power stages (right *y* axis). **b** Power growth and efficiency are represented by red and blue curves, respectively (left *y* axis)

Firstly, we experimentally investigate how variations in the LR linewidth impact the performance and characteristics of few-mode fiber oscillator. Experimental results of the few-mode fiber oscillator constructed with a piece of 45 m commercial 30/600 μm Yb-doped fiber (YDF) and home-made gratings are presented in Fig. 4. The numerical aperture (NA) of the fiber core is ~0.062 with an absorption coefficient of 0.42 dB/m@915 nm. The linewidth of the used HR grating is 4.0 nm with its maximum reflection exceeding 99%. The linewidth of the used LR grating is ~0.2 nm with a reflection of ~5%. The fiber is coiled in a racetrack water-cooled sink, with a minimum bending diameter of 14.5 cm and a maximum of 24.5 cm. The spectra and output powers of the signal laser of the fiber oscillator are respectively displayed in Fig. 4a, b, respectively. At the maximal power, the 3-dB spectral linewidth of the laser is ~2.8 nm and the optical-to-optical (O-O) efficiency is ~78%. The broadening of the spectrum with increasing power can predominantly be attributed to intermodal four-wave mixing^18^.

**Fig. 5:**
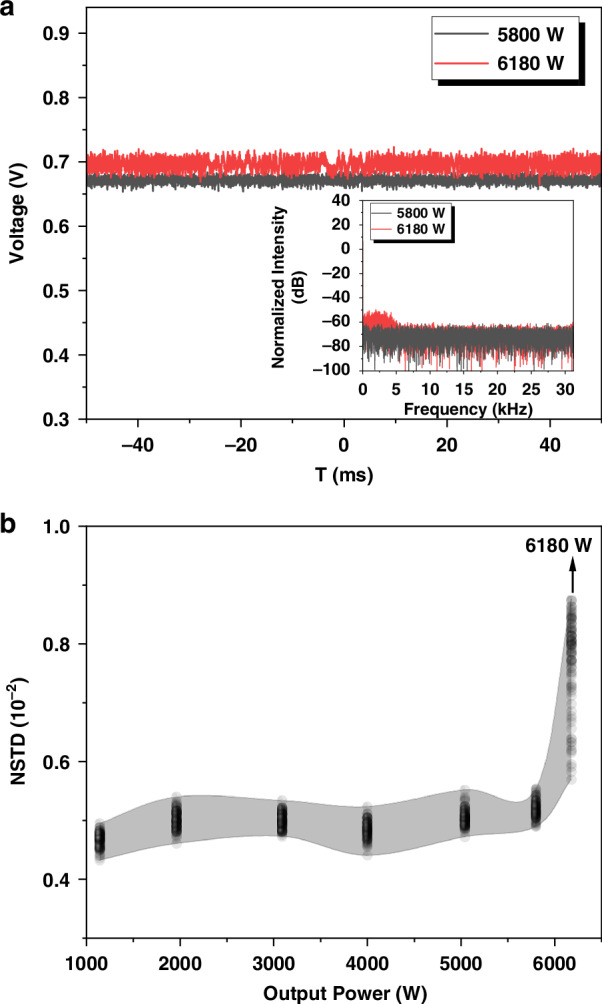
**Temporal frequency characteristics of the fiber oscillator with 0.2-nm LR.** **a** Temporal signals at output powers at 5180 W (black line) and 6180 W (red line), the inset displays the FFT of the temporal signals. **b** NSTD of the temporal signals

The TMI threshold of the oscillator is identified as the power level when the oscillator begins to exhibit evident dynamic changes of beam profile. These changes could be further characterized by the pronounced amplitude noise frequencies resulting from the interference among core modes^40^. The temporal fluctuations below and above the threshold power and their corresponding power spectrum, obtained via fast fourier transform are shown in Fig. 5a. At ~4 kHz, a distinct amplitude noise frequency is observable in the red line of Fig. 5a, indicating that the onset of TMI occurs at the power of 6180 W. Additionally, the low-frequency fluctuations attributed to TMI can also be quantitatively assessed using the normalized standard deviation (NSTD) of the temporal power signal^41^. By examining the photodetector (PtD) signal at different power levels, the corresponding NSTD is plotted as black dots in Fig. 5b. A sharp rise in NSTD, along with increased scatter of the data, is observed when the power reaches 6180 W, confirming the TMI threshold at this power level.

**Fig. 6:**
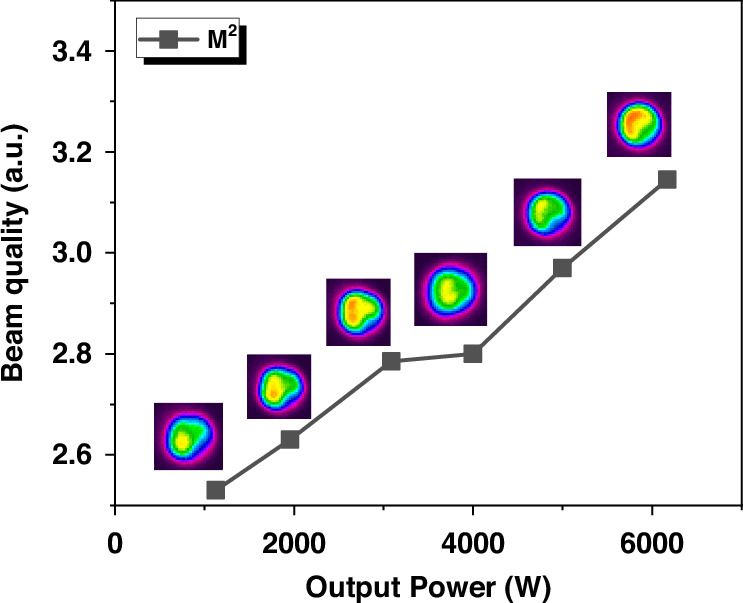
Power-dependent beam quality evolution in a few-mode fiber oscillator with 0.2-nm LR. Beam quality of the fiber oscillator with 0.2-nm LR

The evolution of the beam quality factor (M^2^) for this few-mode fiber oscillator in relation to the output power is depicted in Fig. 6. The M^2^ beam quality gradually increases from 2.53 to 3.15 as the power is scaled from 1138 W to 6180 W. This increase in power is accompanied by a corresponding evolution of the beam profile indicating the growing in the proportion of LP_11_ mode.

**Fig. 7:**
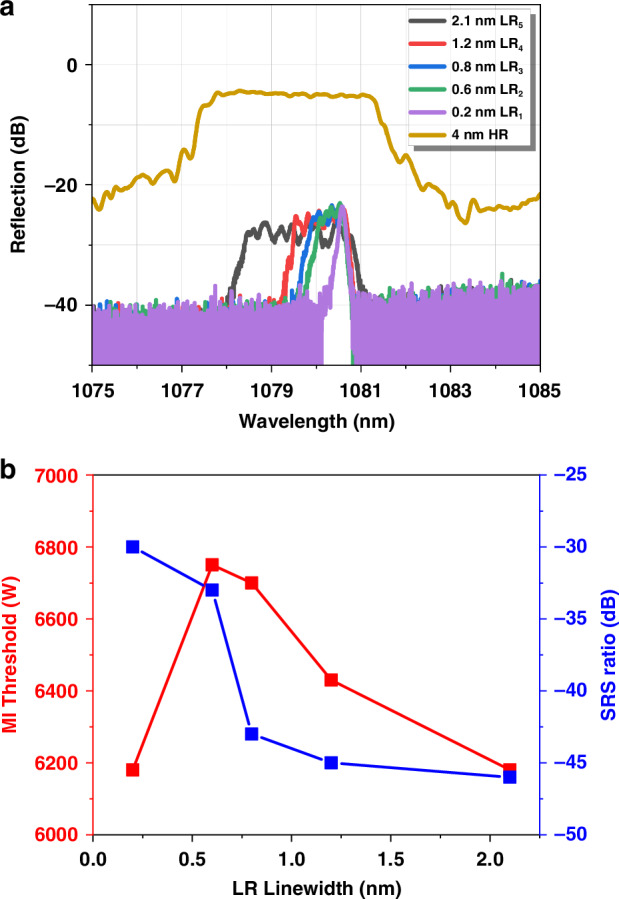
**LR used in fiber oscillator with varying linewidth.** **a** Spectra of LR with linewidth of 2.1 nm (black line), 1.2 nm (red line), 0.8 nm (blue line), 0.6 nm (green line), 0.2 nm (purple line), HR with linewidth of 4 nm (brown line); **b** TMI threshold (left *y* axis) and SRS ratio (right *y* axis)

To elucidate the effect of LR linewidth on the performance of the fiber oscillator, different LR gratings with varying linewidths were employed. The spectra of these gratings are displayed in Fig. 7a, b presents the TMI threshold of the fiber oscillator as a function of the 3-dB linewidth of LR (red line). The fiber oscillator reaches its TMI threshold (6750 W) when using the LR with the 3-dB linewidth of 0.6 nm. Deviations from the linewidth of LR, either narrower or wider (than 0.6 nm), result in a lower TMI threshold.

Our theoretical analysis reveals that, as the linewidth of LR increases and remains relatively narrow, the frequency response of every mode will incrementally broaden, thereby increasing the TMI threshold. However, when the linewidth is too large, the distinct frequency responses of different modes begin to overlap with each other, TMI tends to occur in the coincident region of different modes, resulting in a lower threshold. The blue line in Fig. 7 is the SRS ratio@6 kW varied with LR linewidth. Contrary to the TMI threshold, the SRS ratio monotonically decreases with the growth of LR linewidth. Hence, considering the joint combined inhibition of SRS and TMI, the linewidth of 0.8 nm is suitable for both a high TMI threshold and a low SRS ratio.

**Fig. 8:**
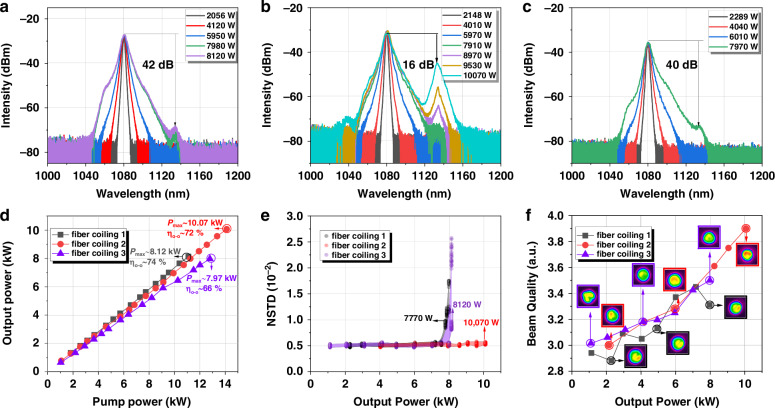
**Performance of fiber oscillator subjected to various bending diameters.** **a** spectra with the minimal bending diameter of 19.5 cm; **b** spectra with the minimal bending diameter of 17.5 cm; **c** spectra with the minimal bending diameter of 14.5 cm; **d** power growing curves with three fiber coiling, with minimal bending diameter of 19.5 cm (black line/dots), 17.5 cm (red line/dots) and 14.5 cm (purple line/dots) ; **e** NSTD of temporal signal; **f** beam quality value with corresponding beam profile under different power stage

Building on our experimental investigation into the effect of LR linewidth on properties of few-mode fiber oscillator, we have uncovered correlations between the spectral properties of the gratings and the TMI threshold of the few-mode oscillator. These findings provide a crucial understanding of how grating characteristics could influence the performance of the few-mode oscillator. Furthermore, we aim to elucidate how TMI threshold varies in the presence of mode-dependent attenuation. In the experiments, another piece of low-NA (0.058) 30/600 μm YDF is used with a length of 34 m, with its absorption coefficient of 0.37 dB/m@915 nm. The linewidth of the HR grating used is still 4.0 nm, while the linewidth of the LR grating used is selected to be ~0.8 nm for a relatively high TMI threshold. A much larger bending diameter was selected, ranging from a minimum of 19.5 cm to a maximum of 28.5 cm. The output power and spectral characteristics of the signal laser in this fiber oscillator are illustrated in Fig. 8a, d, respectively. At the maximum power, the 3-dB linewidth of the laser is ~6.0 nm and the corresponding O-O efficiency is ~74%.

As shown in Fig. 8e, the TMI threshold is approximately 8120 W, corresponding to dots where NSTD become dispersive. The evolution of the M^2^ beam quality factor for this few-mode fiber oscillator, as a function of the output power, is presented in Fig. 8f. The M^2^ beam quality gradually grows from 3.05 to 3.50 with the power scaling from 1104 W to 8120 W.

To probe the influence of mode loss further, we coiled the YDF tighter with the minimum bending diameter of 17.5 cm. The corresponding experimental outcomes are displayed in Fig. 8b. Under this condition, the 3-dB spectral linewidth of the laser at the highest power (10.07 kW) is ~7.3 nm and the corresponding O-O efficiency is ~72%. The dots of NSTD exhibit concentrated patterns, suggesting the absence of significant TMI in this configuration. Namely, the TMI threshold is elevated by decreasing bending radius.

Nonetheless, as the minimal bending diameter is further decreased to 14.5 cm, the TMI threshold instead exhibits a decline. As depicted in Fig. 8e, the TMI threshold can be estimated as 7770 W based on the dispersive data points of NSTD. When at the maximum power, the 3-dB linewidth of the output laser is ~5.5 nm and the SRS ratio is as low as 40 dB (Fig. 8c), where SRS-induced mode coupling could be basically ignored. With the tighter bending, the M^2^ beam quality is lowered to 3.31 at the highest power, as exhibited in Fig. 8f.

**Fig. 9:**
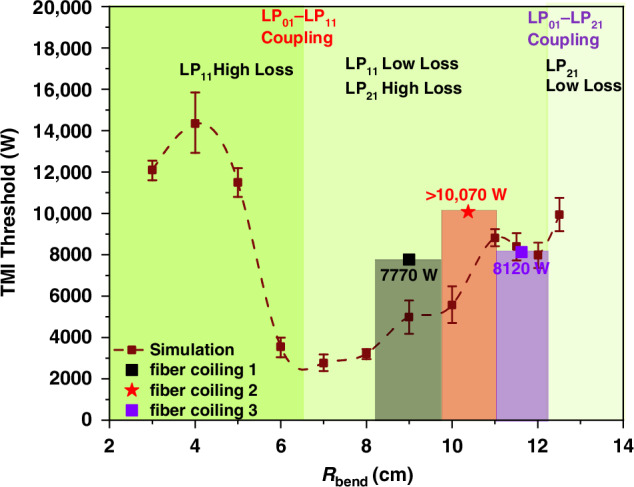
**TMI threshold comparison between simulation and experimental results.** The simulated TMI threshold varies with different bending radii (dark red dashed line with error bars), accompanied by the experimental results with minimal bending diameters of 19.5 cm (black dot/cube), 17.5 cm (red dot/cube), and 14.5 cm (purple dot/cube)

Figure 9 reveals a nonmonotonic relationship between TMI threshold and the bending radius, aligning with the simulation results (dashed line). This implies that fiber bending radius should be properly selected to manage the mode proportion and achieve a relatively high TMI threshold for few-mode fiber oscillator.

## Discussion

In fact, the latest record power of fiber oscillator was reported in 2020 as 8-kW level^4^, whose power promotion is obstructed by SRS. To circumvent the pronounced nonlinear effect, our research team utilized the fiber with an enlarged mode area and also achieved ~8 kW in 2023^38^. Nevertheless, the further power scaling of the fiber oscillator still encountered limitations due to TMI. To mitigate TMI, our research team theoretically examines the relationship between spectral power redistribution of different modes in the fiber oscillator and mode-dependent spectral responses of gratings as well as bending loss. Guided by the simulation results, we selected the low-NA fiber and tried different bending radii experimentally (due to experimental conditions and time constraints, we only attempted three different radii of fiber coiling).

Ultimately, the 10-kW level fiber oscillator is developed and new record power of single-stage monolithic fiber oscillator is achieved to the best of our knowledge. Despite these advancements, the fiber oscillator is operated at few-mode state, which is the next challenge that needs to be tackled. In the future, our research team intends to further optimate the fiber bending and pay more attention to the mode selection in the fiber oscillator to achieve a maintenance-free and robust operation with superior beam quality.

In conclusion, this manuscript presents a detailed theoretical and experimental study of TMI in a few-mode fiber oscillator, particularly focusing on the effects of modal power redistribution in the frequency domain caused by mode-dependent spectral responses and fiber bending loss. Our theoretical analysis reveals a non-monotonic relationship between the TMI threshold and the linewidth of LR and the bending radius. The findings suggest that the different frequency responses of the LR can promote the amplification of new mode components in uncoupled frequency regions, leading to a redistribution of modal power in frequency space. Additionally, the experimental validation indicates that, to achieve a high TMI threshold, both the LR linewidth and bending radius must be appropriately selected, aligning with the theoretical predictions. By optimizing these parameters of fiber oscillator, we have managed to achieve a laser output of 10.07 kW without significant TMI, setting a new record for single-cavity all-fiber oscillators. We wish that our theoretical and experimental analysis on the modal power redistribution in the frequency domain, which is regulated by both mode-dependent spectral responses and fiber bending loss, would provide novel insights and potential methods for mitigating TMI in high-power fiber lasers. We also believe this design of compact 10-kW level all-fiber oscillator could provide references for improving the performance of the fiber laser.

## Materials and methods

### Mode profiles

**Fig. 10:**
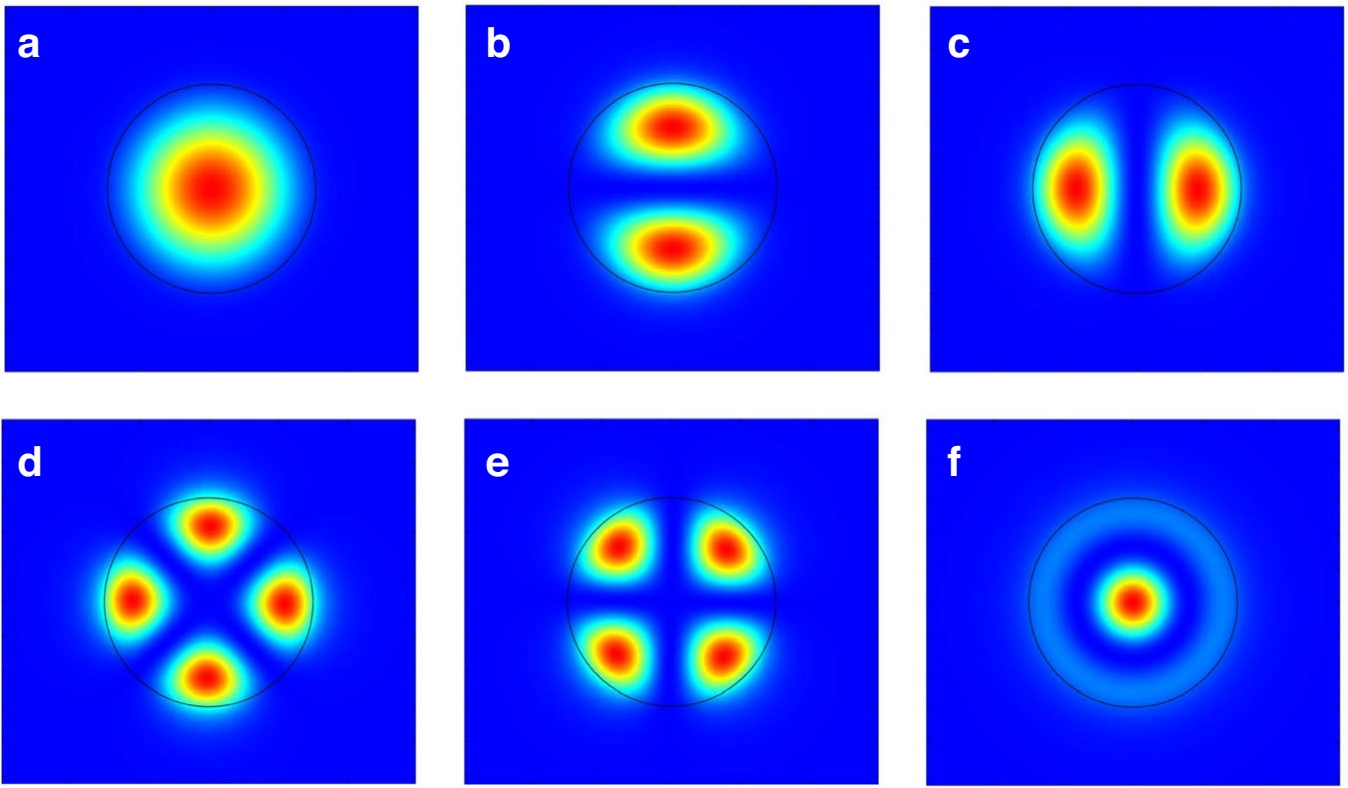
**Power distribution of fiber core modes.** **a** LP_01_; **b** LP_11e_; **c** LP_11o_; **d** LP_21e_; **e** LP_21o_; **f** LP_0_

We consider the typical modality of double-cladding fibers (DCFs) consisting of a core, an inner cladding, an outer cladding and air surrounding. Taking the standard structure of 30/600 μm commercial DCFs for reference, six core modes are allowed to transmit, including the LP_01_ (Fig. 10a), and LP_11e/o_ (Fig. 10b, c), LP_21e/o_ (Fig. 10d, e), and LP_02_ (Fig. 2f) core modes.

### Modal spectral responses of FBGs

Having considered the supported modes, in the simulation of fiber laser, the boundary condition is also vital for differential evolution of laser amplification. During the laser oscillation in the resonant cavity, the boundary conditions are defined as the spectra of the FBGs, where the reflectivities R_HR_ and R_LR_ are simulated based on coupling coefficient calculation^42^ and coupled mode theory^43^. Due to the modal dispersion, every fiber mode has a specific resonant Bragg wavelength:

1 $${\lambda }_{m}=2{n}_{m}\Lambda (1+F)$$

Table 1 lists the corresponding resonant wavelength of every mode for a grating period Λ of 372.1 nm. The wavelength interval between LP_01_ and LP_11_ is ~0.22 nm. It could be permitted that modes would own a relatively independent region when linewidth of single mode is smaller than ~0.4 nm.

**Table 1:** Parameters of FBGs

Mode/parameter	Resonant wavelength (nm)	LR reflectivity (%)	HR reflectivity (%)
LP_01_	1079.96	~9.9	>99.5
LP_11e_	1079.74	~8.9	~99.0
LP_11o_	1079.74	~8.9	~99.0
LP_21o_	1079.49	~7.8	~98.3
LP_21e_	1079.49	~7.8	~98.3
LP_02_	1079.40	~7.0	~97.5

**Fig. 11:**
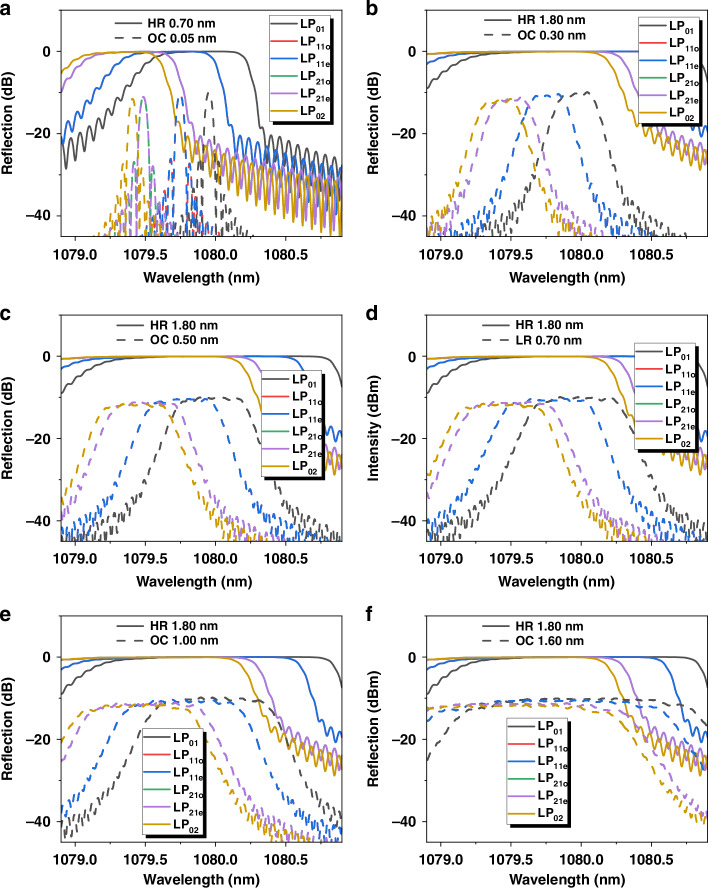
**Reflection Spectra of FBGs with different linewidths.** **a** HR/LR with the 3-dB linewidth of 0.7/0.05 nm; **b** HR/LR with the 3-dB linewidth of 1.8/0.3 nm; **c** HR/LR with the 3-dB linewidth of 0.5 nm; **d** HR/LR with the 3-dB linewidth of 0.7 nm; **e** HR/LR with the 3-dB linewidth of 1.8/1.0 nm; **f** HR/LR with the 3-dB linewidth of 1.8/1.6 nm

In simulations, several linewidths of the LR FBGs are selected from 0.05 to 2.0 nm, while the linewidths of the HR FBGs are set to 0.7 and 1.8 nm, respectively. For the simulated spectra (Fig. 11), it widens with the linewidth of the reflection spectrum to 0.3 nm (Fig. 11b), and the reflection wavelength of LP_01_ and LP_11_ modes begins to emerge with each other. As the linewidth of the simulation spectra increases further, the reflection region of LP_11_ and LP_21_ modes starts to overlap. When the linewidth reaches ~0.7 nm (Fig. 11d), resonant region of LP_01_ mode begins to have common part with LP_21_ mode. The modal specific responses of FBGs largely determines the overlap of mode component in some wavelength regions, where the mode coupling is more likely to occur. It also has been confirmed that the linewidth of the HR FBG has little influence on the TMI threshold. Apart from deciding the boundary condition of the laser oscillation, the spectral reflectivities of the FBGs influences the amplification of all modes. However, mode loss should contribute much more to the modal amplification directly.

### Effective bending radius

**Fig. 12:**
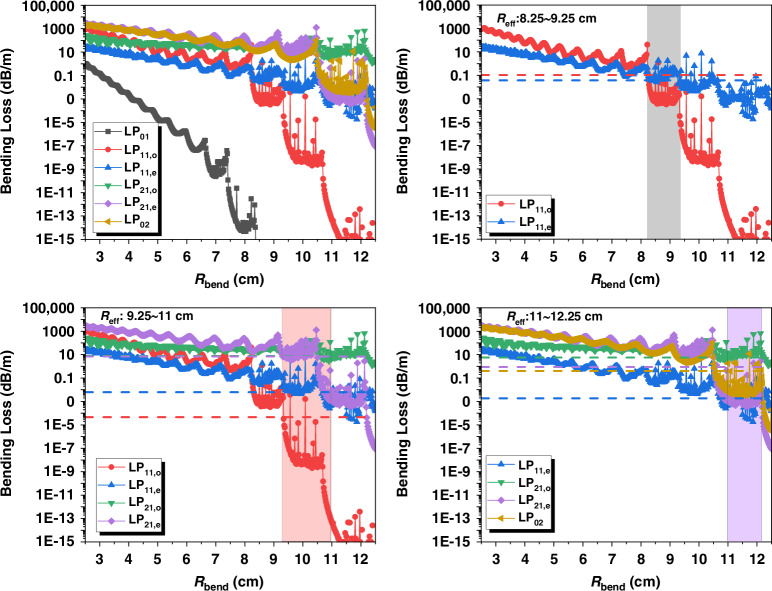
**Simulated mode loss.** **a** The value of mode loss varies with bending radius. **b** Effective bending radius estimation of fiber coiling 1. **c** Effective bending radius estimation of fiber coiling 2. **d** Effective bending radius estimation of fiber coiling 3

According to the analytical theory model of mode loss^44,45^, the bending losses *α*_*m*_ of six supported modes in 30/600 μm fiber were computed. Figure 12a illustrates that the bending loss values of six modes as functions of bending radius are plotted as different colored symbols. From the results, minor but frequent fluctuations could be seen in bending loss of every mode. By using the approximate analytical solution, the alternating weight of the bending loss could be directly conducted, which makes the bending loss not linearly reduce with bending radius but have distinct stepped shape. Therefore, at these stepped reductions, the bending loss is not sensitive to the bending radius. However, if the bending loss is not at the plateau position of the curve, it would face a sudden drop with a slightly bigger fiber coiling, which indicates that the TMI threshold will also have intervals of abrupt change.

In contrast to theoretical predictions, in the practical experiments, the racetrack coiling of fiber is used, which means the bending loss is varying with the coiling of the gain fiber. The effective bending radius can be inferred from the overlapping section of effective bending loss *α*_*m,eff*_ and *α*_*m*_, where *α*_*m,eff*_ could be determined by the integral mean value:

2 $${\alpha }_{m,eff}=\frac{{\int }_{0}^{L}{\alpha }_{m}(l)dl}{L}$$

Therefore, the effective bending radii of the experiments could be estimated by interleaved curves shown in Fig. 12b–d.

### Gain saturation and thermally induced coupling

The net gain, which is crucial for power amplification of laser modes, is related to both mode loss and gain. During mode competition, gain saturation is a vital remoting mechanism. Considering the gain saturation^39^, the total saturated gain distribution could be expressed as:

3 $$g({\bf{r}})=\frac{{g}_{0}}{1+{I}_{s}({\bf{r}})/{I}_{sat}}$$

where *I*_*s*_ is the light intensity of the signal light, the saturated light intensity *I*_*sat*_ could be expressed as:

4 $${I}_{sat}=[{P}_{p}(z)({\sigma }_{p}^{a}+{\sigma }_{p}^{e})/({E}_{p}{A}_{clad})+1/\tau ]\frac{{E}_{s}}{{\sigma }_{s}^{a}+{\sigma }_{s}^{e}}$$

and the gain coefficient *g*_0_ could be expressed as:

5 $${g}_{0}(z)=\frac{{I}_{p}(z)({\sigma }_{p}^{a}{\sigma }_{s}^{e}-{\sigma }_{p}^{e}{\sigma }_{s}^{a})/{E}_{p}-{\sigma }_{s}^{a}/\tau }{{I}_{p}(z)({\sigma }_{p}^{a}+{\sigma }_{p}^{e})/{E}_{p}+1/\tau }{N}_{0}$$

where *I*_*p*_ is the pump light intensity; ${\sigma }_{p}^{a}$ and ${\sigma }_{p}^{e}$ are the absorption and emission cross-sections of pump wavelengths, respectively; ${\sigma }_{s}^{a}$ and ${\sigma }_{s}^{e}$ are the absorption and emission cross-section of signal wavelength, respectively; *E*_*p*_ and *E*_*s*_ are the energies of photon at pump and signal wavelength. Using rate equation, the saturated gain could be expressed as the product of the average power gain saturation term and the normalized gain saturation distribution term:

6 $$g({\bf{r}})=\tilde{g}(z)\frac{{\delta }_{ave}}{{\delta }_{dis}}$$

In Eq. 6, the average power gain saturation coefficient ${\delta }_{{ave}}=1+{I}_{s0}/{I}_{{sat}}$ and the gain saturation coefficient ${\delta }_{{dis}}=1+{I}_{s}/{I}_{{sat}}$ represents the degree of gain variation caused by horizontal distribution of power, where *I*_*s*0_ is the average light intensity of signal:

7 $${I}_{{s}0}=\frac{{P}_{s}(z)}{{A}_{eff}}$$

If omitting the relaxation term, *δ*_*ave*_ could be expressed as:

8 $${\delta }_{ave}=1+\frac{{I}_{s0}}{{I}_{sat}}\approx 1+\frac{{A}_{clad}({\sigma }_{as}+{\sigma }_{es})}{{A}_{eff}({\sigma }_{ap}+{\sigma }_{ep})}$$

Import the parameters from Table 2 into Eq. 8. It could be conducted that $\delta \gg 1$. Therefore, the saturated gain could be simplified as:

9 $$\tilde{g}(z)=\frac{{g}_{0}}{{\delta }_{ave}}\approx \frac{{A}_{eff}({\sigma }_{ap}+{\sigma }_{ep})}{{A}_{clad}({\sigma }_{as}+{\sigma }_{es})}{g}_{0}$$

**Table 2:** Parameters of doped fiber

Fiber Parameters	LMA-DCF-30/600
core-to-cladding area ratio (~*A*_eff_/*A*_clad_)	2.4 × 10^−3^
Absorption cross-section@1080 nm (*δ*_*as*_)	2.28 × 10^−27^ (m^2^)
Emission cross-section@1080 nm (*δ*_*es*_)	2.82 × 10^−25^ (m^2^)
Absorption cross-section@982 nm (*δ*_*ap*_)	6.21 × 10^−25^ (m^2^)
Emission cross-section@982 nm (*δ*_*ep*_)	8.19 × 10^−25^ (m^2^)

Moreover, the gain of different modes could be computed by calculating the confined coefficient:

10 $${\Gamma }_{m}={\int }_{0}^{2\pi }{\int }_{0}^{{r}_{core}}{\psi }_{m}{\psi }_{m}^{\ast }r\frac{{\delta }_{ave}}{{\delta }_{dis}}{\rm{d}}r{\rm{d}}\theta$$

Thus, the gain of the *m*^th^ mode could be conducted as:

11 $${\tilde{g}}_{m}={\Gamma }_{m}\tilde{g}(z)$$

With the boundary conditions and gain saturation having been analyzed, the process of thermally-induced mode coupling, which would significantly influence the efficiency and power ratio of modes in the oscillator, could be modeled.

The thermally-induced mode coupling could be described by the photothermal coupled equation^12,13,15^:

12 $$\frac{\partial {P}_{m}}{\partial z}=({\tilde{g}}_{m}-{\alpha }_{m}){P}_{m}+\tilde{g}(z){P}_{m}\sum _{n\ne m}{\chi }_{mn}{P}_{n}$$

where *P*_*m*_ is the module of normalized amplitude (*m* for the *m*^th^ mode):

13 $${P}_{m}\approx {\left|\sqrt{\frac{{n}_{1}{\xi }_{0}c}{2}}{A}_{m}\right|}^{2}$$

The amplitude of *m*^th^ mode is defined as *A*_*m*_; *ε*_0_ is the dielectric constant in vacuum and *c* is the light velocity. The mode gain is defined as ${\widetilde{g}}_{m}$, which is calculated by modal gain overlapping integral. The thermally induced coefficient ${{\chi }}_{{mn}}$ is calculated by overlap integral of thermally-light coupling, respectively. The mode loss *α*_*m*_ is calculated in part B.

### Experimental setup

This section delineates the experimental setup in detail, highlighting its critical elements and the reasons behind their selection to ensure the accuracy and reliability of our experimental results.

**Fig. 13:**
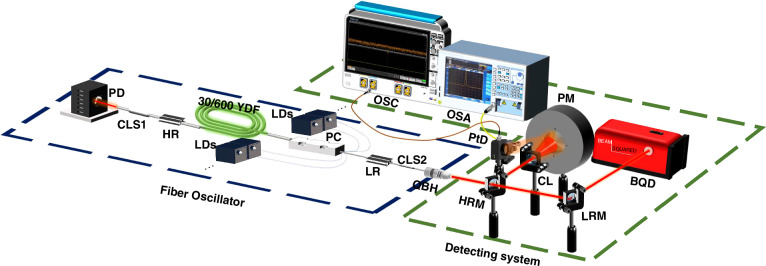
**Experimental setup of a few-mode fiber oscillator.** The setup is comprised of the fiber oscillator (blue dashed box) and the detecting system (green dashed box)

The overall experimental setup is shown in Fig. 13, which consists of two principal components, including the few-mode fiber oscillator and the detecting system. The resonant cavity of fiber oscillator is composed of a piece of 30/600 μm YDF and a pair of home-made FBGs, specifically a LR and a HR pair. A cladding laser stripper (CLS)1 is affixed to the HR pigtail to eliminate the extraneous cladding laser. Subsequently, the backward-propagating laser that passes through CLS1 would be captured by a power detector. The YDF is wound into racetrack-shaped groove with a 20-cm straightway in the water-cooled plate. The pump power of the oscillator is provided by wavelength-locked (central wavelength @969 & 982 nm) laser diodes, which are connected by a pump combiner (PC). The PC is placed in the resonant cavity between YDF and LR. The signal laser, selectively filtered by FBGs, is amplified by the pump power injected by YDF. The amplified laser coming out of the oscillator would be stripped of the unwanted cladding laser by CLS2. The detecting system would then record the light emitted through the quartz block head (QBH). Firstly, most of the laser transmitted through QBH is reflected by a high-reflection mirror and subsequently expanded by a concave lens before being captured by the power meter (PM) to measure the output power of the oscillator. The transmitted light would be reflected by a low-reflection mirror and enter the beam quality detector to evaluate the laser beam profile. Additionally, a minor portion of the laser would be reflected by the absorbing surface of PM and channeled into the optical spectrum analyzer via a fiber jumper to analyze the spectral characteristics. Lastly, a part of the reflected laser would be captured by a PtD to identify the temporal stability of TMI.

## Data Availability

The data underlying the results presented in this paper are not publicly available at this time but may be obtained from the authors upon reasonable request.
